# An Overview of Two-Component Signal Transduction Systems Implicated in Extra-Intestinal Pathogenic *E. coli* Infections

**DOI:** 10.3389/fcimb.2017.00162

**Published:** 2017-05-09

**Authors:** Erin J. Breland, Allison R. Eberly, Maria Hadjifrangiskou

**Affiliations:** ^1^Department of Pharmacology, Vanderbilt University Medical CenterNashville, TN, USA; ^2^Department of Pathology, Microbiology and Immunology, Vanderbilt University Medical CenterNashville, TN, USA; ^3^Department of Urology, Vanderbilt University Medical CenterNashville, TN, USA

**Keywords:** APEC, ExPEC, MAEC/NMEC, UPEC, two-component systems, signal transduction, virulence factors

## Abstract

Extra-intestinal pathogenic *E. coli* (ExPEC) infections are common in mammals and birds. The predominant ExPEC types are avian pathogenic *E. coli* (APEC), neonatal meningitis causing *E. coli*/meningitis associated *E. coli* (NMEC/MAEC), and uropathogenic *E. coli* (UPEC). Many reviews have described current knowledge on ExPEC infection strategies and virulence factors, especially for UPEC. However, surprisingly little has been reported on the regulatory modules that have been identified as critical in ExPEC pathogenesis. Two-component systems (TCSs) comprise the predominant method by which bacteria respond to changing environments and play significant roles in modulating bacterial fitness in diverse niches. Recent studies have highlighted the potential of manipulating signal transduction systems as a means to chemically re-wire bacterial pathogens, thereby reducing selective pressure and avoiding the emergence of antibiotic resistance. This review begins by providing a brief introduction to characterized infection strategies and common virulence factors among APEC, NMEC, and UPEC and continues with a comprehensive overview of two-component signal transduction networks that have been shown to influence ExPEC pathogenesis.

## Introduction to extraintestinal pathogenic *E. coli* (ExPEC)

Since the discovery by Theodor Escherich in 1885, *Escherichia coli* has become one of the most tractable model organisms for study and utilization in the lab. For this reason, numerous studies use laboratory strains of *E. coli* for comparative and analytical studies, sometimes over-simplifying the complexity and diversity of the *E. coli* species. To date, over 3,600 genomes have been sequenced in part or in full, revealing seven major phylogenetic groups—A, B1, B2, C, D, E, and F—with the remaining unclassified subtypes placed in an eighth group, *Escherichia* cryptic clade I (Herzer et al., 1990; Clermont et al., 2013).

*E. coli* colonize the gastrointestinal (GI) tracts of humans and other warm-blooded mammals, and in this context, they comprise part of the organism's normal flora (Dubos and Schaedle, 1964), or microbiome, as coined by Joshua Lederberg in 2001. However, the acquisition of genetic elements, primarily through horizontal gene transfer, gives rise to several different pathogenic *E. coli* with distinct virulence strategies. Gastrointestinal or diarrhegenic *E. coli* pathotypes include diffusely adherent (DAEC), enteroaggregative (EAEC), enterohemorrhagic (EHEC), enteroinvasive (EIEC), enteropathogenic (EPEC), and enterotoxigenic (ETEC). However, extra-intestinal pathogenic *E. coli* (ExPEC) pathotypes have emerged (Russo and Johnson, 2000), and they include avian pathogenic *E. coli* (APEC), neonatal meningitis causing or meningitis-associated *E. coli* (NMEC/MAEC), and uropathogenic *E. coli* (UPEC). The steady, yet up until recently under-appreciated, rise in antimicrobial resistant *E. coli* has played a significant role in the increasing incidence and lethality of extra-intestinal *E. coli* infections.

**Figure 1:**
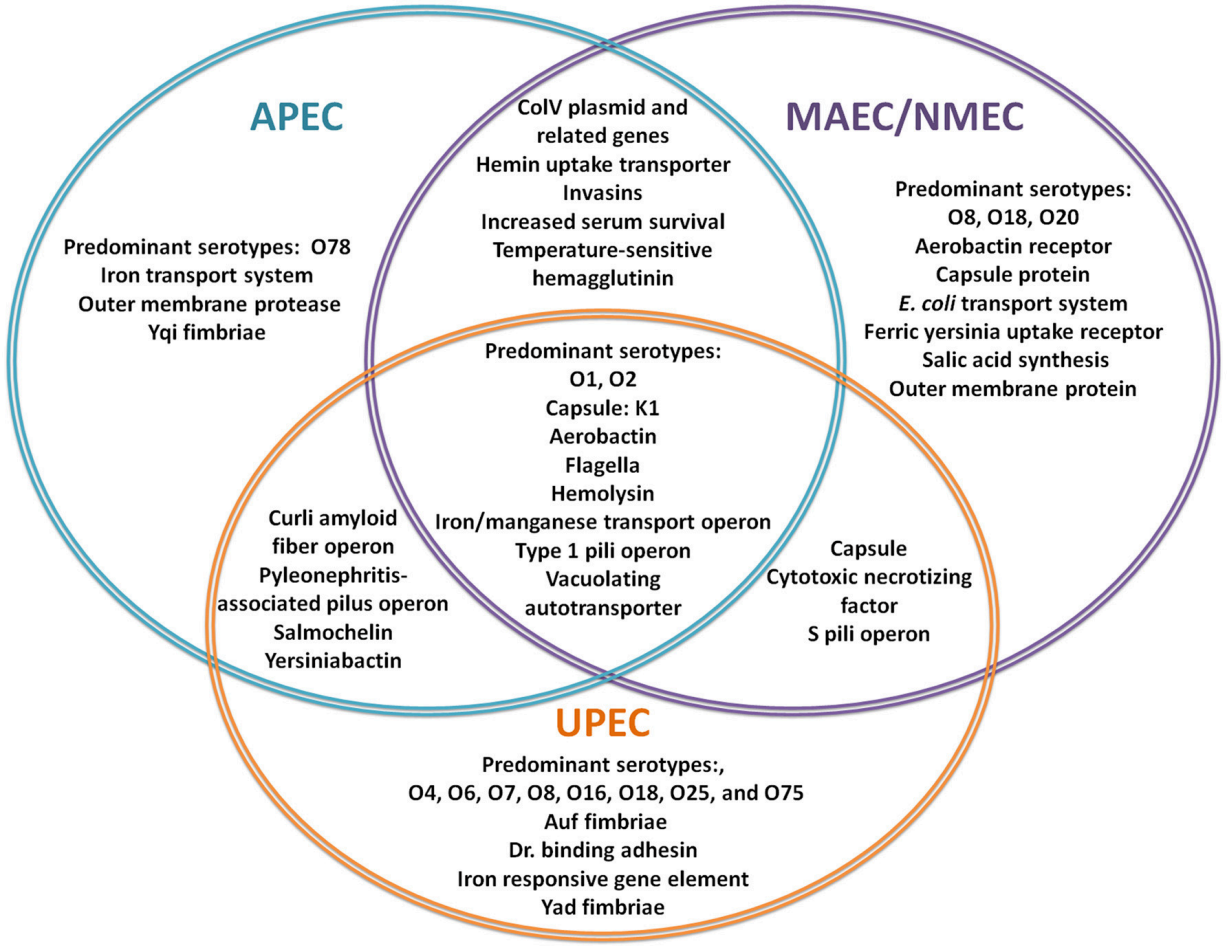
**Virulence factors involved in ExPEC infections**. The Venn Diagram represents the most commonly reported, shared and individual, virulence factors for APEC (blue), MAEC/NMEC (purple), and UPEC (orange). (Knöbl et al., 2001; Johnson et al., 2006; Lloyd et al., 2007; Wiles et al., 2008; Zhu et al., 2010; Nazemi et al., 2011; Spurbeck et al., 2011; Logue et al., 2012; Zhu Ge et al., 2014; Huja et al., 2015; Wang et al., 2015; Wijetunge et al., 2015).

**Figure 2:**
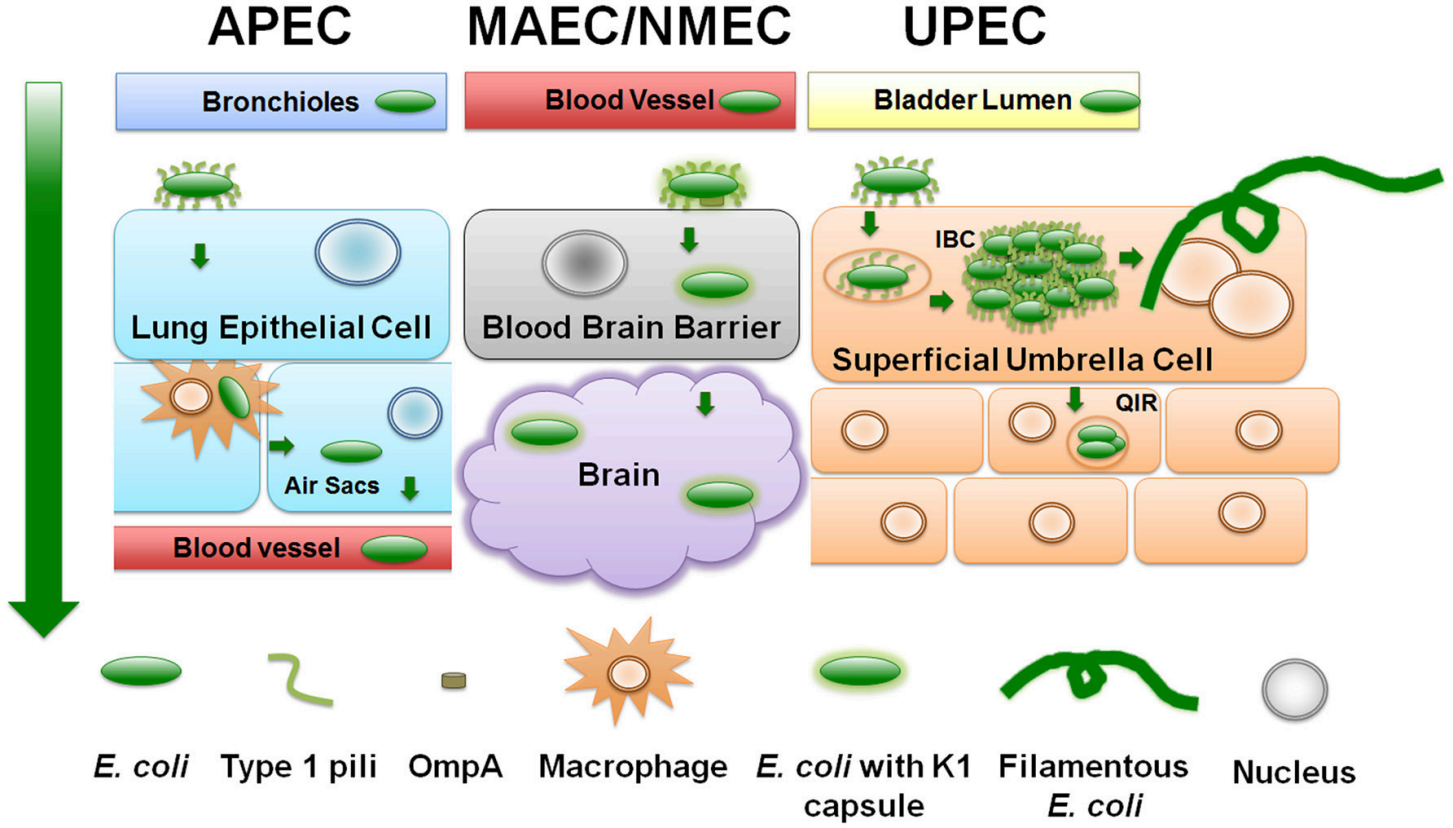
**ExPEC infection strategies**. Diagram depicts a generalized schematic of the known and relevant aspects of ExPEC infections. The leftmost green arrow depicts the typical route of infection from point of entry. APEC attach to upper respiratory epithelial cells using type 1 pili. APEC can replicate and transverse the respiratory tract to the bloodstream by means of avian macrophages. NMEC/MAEC exit the bloodstream and attach via type 1 pili to brain micro-vascular endothelial cells that comprise the blood brain barrier. NMEC enter the endothelial cells through OmpA receptor-mediated entry. From here, NMEC are able to colonize the brain and meninges. UPEC attach to urothelial cells in a type 1 pili-dependent manner. UPEC are then endocytosed and escape into the cytosol where they replicate into intracellular bacterial communities (IBC). UPEC escape the IBC state by filamenting and fluxing out of the infected host cell. Dispersing UPEC can infect neighboring or underlying transitional cells, and/or can ascend the ureters to colonize and infect the kidneys.

**Figure 3:**
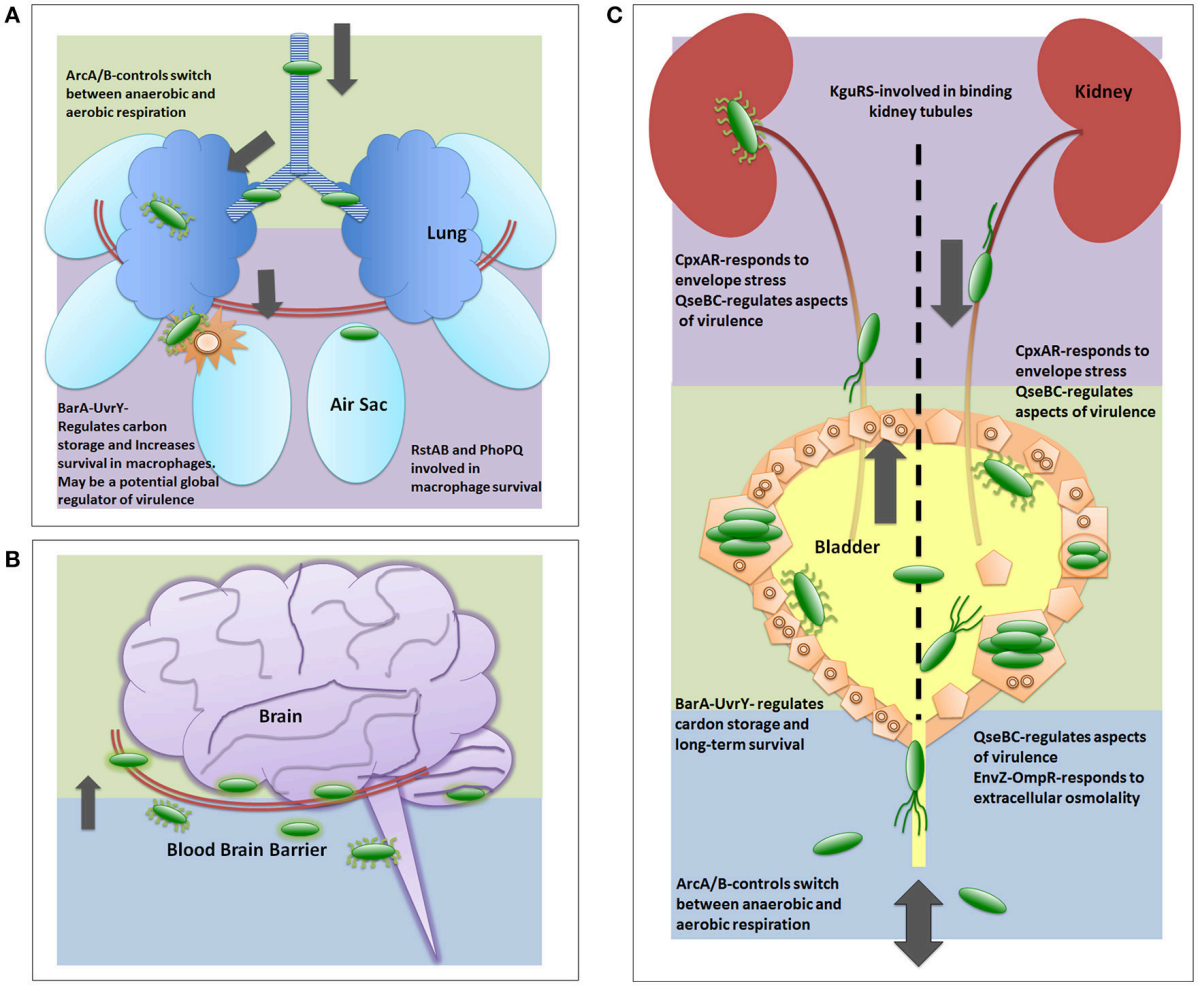
**Two-component systems involved in UPEC pathogenesis**. The two-component systems are listed in the general order in which they are reported as critical for each infection strategy. **(A)** depicts a generalized view of APEC pathogenesis infecting an avian respiratory tract. Early infection is denoted by green color. Late infection is outlined by purple color. **(B)** depicts a generalized view of MAEC/NMEC infection in a human brain by crossing the blood brain barrier. Following bacteria entering the blood stream, early meningitis infection is denoted by blue background where *E. coli* cells bind and traverse the blood brain barrier. Late infection is outlined by green background, which includes infection of the meninges. There are no publications on TCS involved in MAEC/NMEC pathogenesis; however, capsule, pili, and other virulence factors are required for pathogenesis and these are known to be regulated in part by TCS. **(C)** depicts a generalized view of UPEC infecting a human urinary tract. Blue background indicates entry and initiation of UPEC infection. Green depicts infection in the bladder. Purple depicts the ascension into and infection of the kidney.

**Figure 4:**
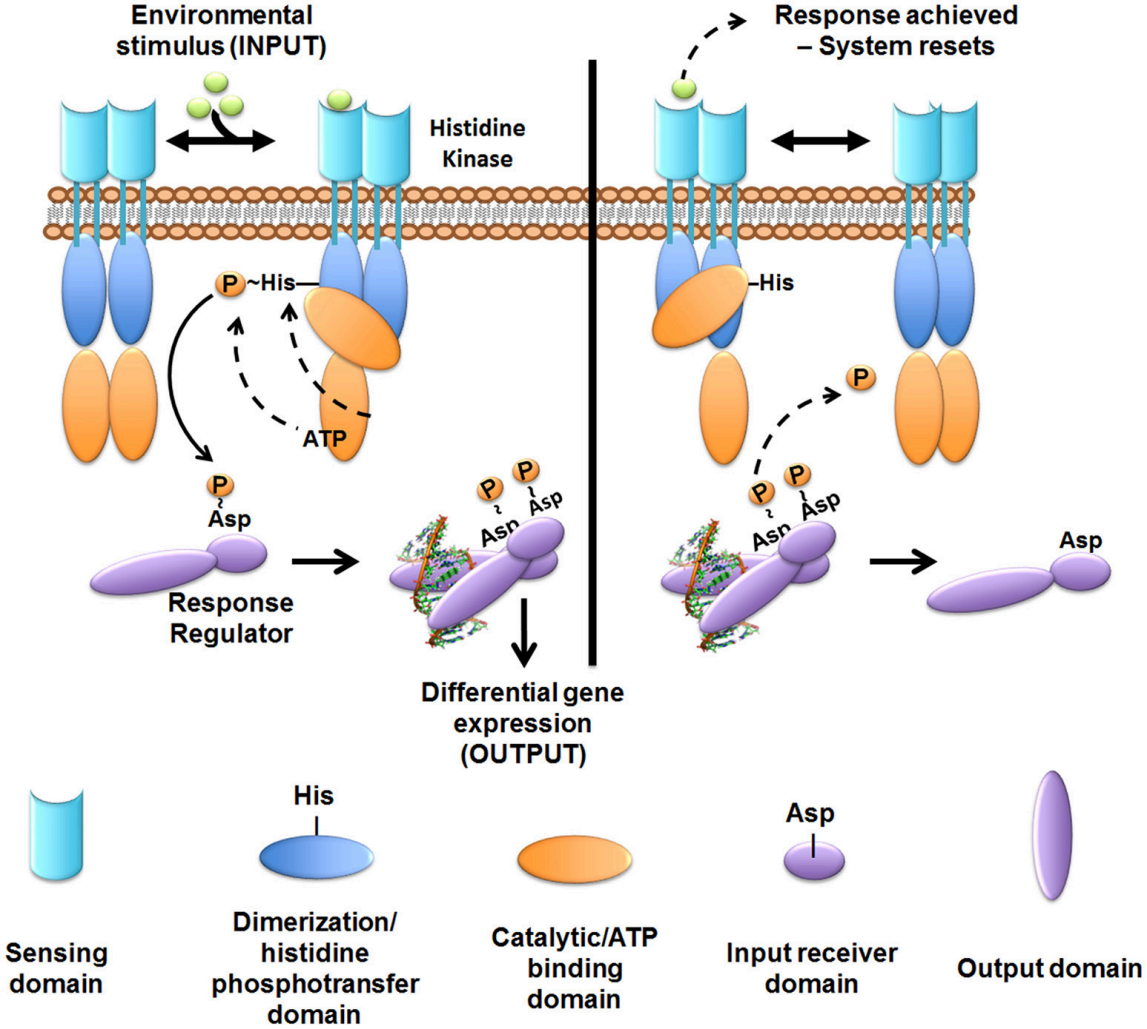
**Overview of two-component system signal transduction**. In most cases studied to date, the sensor histidine kinase is membrane-embedded. The sensor kinase detects signals or stimuli and undergoes auto-phosphorylation at a conserved histidine residue. The phosphoryl-group is then transferred to the cognate cytoplasmic response regulator at a conserved aspartate residue. Phosphorylated response regulators form an active dimer that can then regulate gene transcription. Following the appropriate cellular response, the sensor exhibits phosphatase or reverse phosphotransferase activity removing the phosphoryl-group from the response regulator to “reset” the system. While most kinases are found as a dimer in the membrane, dynamic interactions between the mono- and di-meric state may occur.

The human ExPEC strains predominantly cluster in the B2 and D phylogenetic groups while APEC strains have also expanded into C and F groups (Johnson et al., 2001; Sokurenko et al., 2004; Coque et al., 2008; Nicolas-Chanoine et al., 2008; Totsika et al., 2011). ExPECs colonize and infect a wide range of host species, using an armamentarium of virulence factors that are not restricted to the ExPEC pathotype (Figure 1). The presence of certain combinations of virulence factors can result in extra-intestinal pathogenesis, but among the different ExPEC pathotypes, there is little or no distinct set of virulence factors that is specific to UPEC, APEC, or NMEC. Rather, differential regulation of common virulence factors may be a key driver in the hierarchical expression of specific gene sets that enable/enhance colonization in distinct extra-intestinal niches (Figures 2, 3). As is true for all bacteria, ExPECs deftly respond to environmental stimuli using several signaling networks; the best characterized of these signaling systems are two-component systems (TCSs; Figure 4). This review will outline the infection strategies of APEC, NMEC, and UPEC (Figures 2, 3) and will discuss TCSs that have been shown to contribute to the pathogenesis of these ExPEC pathotypes.

## Diseases in animals and humans caused by ExPECs

Most *E. coli* are commensal bacteria colonizing the gut of many mammals and birds (Jones and Nisbet, 1980); when these bacteria enter sites other than the GI, they may cause disease.

### Disease in birds

Infections by emerging avian pathogenic *E. coli* (APEC) strains (Moulin-Schouleur et al., 2007) cause high morbidity and mortality in flocks and account for considerable economic losses in the poultry industry (de Brito et al., 2003). Interestingly, recent studies show that human consumption of infected poultry meat or eggs can result in food-borne, extra-intestinal diseases in humans (Mitchell et al., 2015; See Section Additional Reservoirs and Research Models: Zoonotic Potential for Urinary Tract Infection), which adds an additional concern for the poultry industry regarding food safety. APEC are the etiologic agent associated with colibacillosis, however severity of disease increases with co-morbid viral infections, such as Newcastle virus and avian infectious bronchitis virus, as well as with bacterial infections by *Mycoplasma gallisepticum* (Merck and Co., 1955). Disease manifestations include colibacillosis, an infection that includes acute fatal septicemia or colisepticemia, sub-acute pericarditis, airsacculitis, salpingitis, and peritonitis (de Brito et al., 2003). Current treatment relies on the use of antibiotics, as well as some commercially available heat-killed vaccines (Merck and Co., 1955).

### Disease in household pets

Cats and dogs are susceptible to urinary tract infections (UTIs) and recurrent UTIs (Johnson et al., 2001; Hutchins et al., 2014). In dogs, ExPEC strains are most commonly associated with uncomplicated UTIs, but have also been the cause of pyometra, mastitis, otitis, prostatitis, bacteremia, skin diseases, cholecystitis, and pneumonia (Oluoch et al., 2001; Ewers et al., 2014). Interestingly, while cats experience idiopathic lower UTI symptoms, no known association with UPEC or UPEC-like strains has been established to date (Bell and Lulich, 2015). However, bronchopneumonia caused by *E. coli* harboring α-hemolysin and cytotoxic necrotizing factor (CNF) has also been reported in cats and dogs (Handt et al., 2003; Sura et al., 2007). Finally, recent reports have documented UTIs in big cats such as snow- and black-leopards, indicating that animals in captivity (such as zoo animals), and potentially in the wild, are susceptible to infections by ExPEC (Carvalho et al., 2012).

### Disease in large ruminants and domestic farm animals (cattle, horses, pigs)

Studies have shown that calves and cows are both likely to develop UTIs, and *E. coli* is the most predominant etiologic agent (Yeruham et al., 2006). UTI symptoms in cattle include depression, muscle wasting and weakness, reduced feed intake, reduced milk production, and weight loss (Yeruham et al., 2006), all of which impact the dairy and meat industries. Additionally, cows are frequently catheterized to collect total urine for nutritional analyses. This repetitive catheterization results in increased risk for ascending UTIs (Tamura et al., 2014). In addition to UTIs, another costly disease in cows is clinical mastitis (Rollin et al., 2015). Clinical mastitis in an inflammation of the udders due to blockage or infection that results in visually abnormal milk production (Thompson-Crispi et al., 2014). The mammary pathogenic *E. coli* or MPEC are predominant bacteria in clinical mastitis (Shpigel et al., 2008).

Horses have been reported to have both hemorrhagic pneumonia and soft tissue ExPEC infections (DebRoy et al., 2008; Ewers et al., 2014). Clinical symptoms of infection include animals lying on their side, abdominal breathing, shaking, convulsion, lameness, and death (Liu et al., 2015). Recently, Liu et al. (2015) performed the first genomic analyses of a porcine-specific ExPEC, PCN033, isolated from the brain of a pig (suggesting a meningitis-causing isolate; Tan et al., 2011). The genomic data placed PCN033 in the D phylogeny group and studies using an ear vein piglet (4–5 weeks) infection model demonstrated the pathogenic potential of this strain (Liu et al., 2015).

### Disease in humans

The most common diseases caused by ExPEC in humans include neonatal meningitis, UTIs, sepsis, pneumonia, and surgical site infections (Riley, 1972; Russo and Johnson, 2003; Kaper, 2005; Mellata, 2013). NMEC is the leading Gram-negative cause of neonatal meningitis cases (Kim, 2016), while UPEC strains are responsible for 75–90% of uncomplicated UTIs and are the leading cause of catheter-associated UTIs (Foxman, 2014; Becknell et al., 2015). In addition, UTIs are a leading cause of *E. coli* bacteremia (Jackson et al., 2005; Al-Hasan et al., 2010). The most common clinical symptoms for UTIs, as is reported in college age women, include increased frequency and urgency of urination, abdominal discomfort, dysuria, nocturia, and hematuria (Vincent et al., 2013).

## Virulence strategies in ExPEC: same soldiers, different commanders

Despite the different tissue/host tropism, APEC, NMEC, and UPEC share many common virulence factors (Figure 1). Among these common virulence factors are pili assembled by the chaperone-usher pathway (CUP), protein adhesins, toxins, iron acquisition systems, transport systems, and other non-essential factors (Russo and Johnson, 2000). However, these virulence factors are subject to distinct regulation depending on the host niche that each ExPEC pathotype harbors. Below, is a brief outline of the infection cascades followed by NMEC, APEC, and UPEC utilizing common virulence factors as they have been described using murine models of infection (Section Additional Reservoirs and Research: Murine Model).

### Avian pathogenic *E. coli* (APEC)

Entering through respiratory inhalation, APEC strains use type 1 pili to adhere to and invade epithelial cells lining the pharynx and trachea (Figures 2, 3A; Mellata et al., 2003). In addition to type 1 pili, Yqi pili were identified as another key adhesion factor during initial stages of avian infection of the lung (Kaper, 2005; Antão et al., 2009). P pili are used later during infection to facilitate dissemination to other parts of the lower respiratory tract or into the bloodstream. Besides the three CUP pili above, APEC also utilize curli amyloid fibers during the early steps of infection (Mellata et al., 2003). APEC can replicate within epithelial cells, avian granulocytes called heterophils, and macrophages (Mellata et al., 2003). Subsequent escape from phagocytes allows APEC entry into the bloodstream at sites of gas exchange in the air sacs (Mellata et al., 2003).

In a comparative genomic study, only five virulence factors, an outer membrane protein, AatA, and components of an iron transporter system, EitA-D, were specific to APEC isolates compared to other ExPEC (Figure 1; Zhu Ge et al., 2014). Since APEC and UPEC virulence factors are similar, APEC plasmids in *E. coli* can also contribute to UTI in mice and have been recently associated with food-borne UTIs (Johnson et al., 2006; Nordstrom et al., 2013; See Section Additional Reservoirs and Research: Zoonotic Potential for Foodborne Urinary Tract Infection).

### Neonatal meningitis-causing *E. coli* (NMEC)/meningitis associated *E. coli* (MAEC)

NMEC infection begins with bacteria entering the bloodstream and replicating to levels above 10^3^ colony-forming units (CFU) per milliliter of blood. Bacteria are then able to breach the blood-brain barrier via binding to receptors on brain micro-vascular endothelial cells. Like in APEC and UPEC strains, CUP pili (Waksman and Hultgren, 2009) are critical for NMEC pathogenesis. Specifically, NMEC type 1 pili mediate attachment to the brain epithelial layer and are critical for biofilm formation, as shown in *in vitro* and *in vivo* studies (Yamamoto et al., 1990; Connell et al., 1996b; Martinez et al., 2000; Mellata et al., 2003; Klemm and Schembri, 2004; Mittal et al., 2011). OmpA is another important NMEC virulence factor, aiding in bacterial invasion into brain micro-vascular epithelial cells (Prasadarao et al., 1996). Following pili- and OmpA-mediated adherence, NMEC become internalized in a process mediated by the CNF1 toxin (Figures 2, 3B). Within host cells, the K1 capsule surrounding the bacteria prevents lysosomal fusion and allows NMEC to infect the subarachnoid space of the meninges (Kim et al., 1992; Xie et al., 2004). NMEC can also invade macrophages, using them as Trojan horses to cross the blood brain barrier (Mittal et al., 2010, 2011).

### Uropathogenic *E. coli* (UPEC)

UPEC typically cause ascending infections, entering the urinary tract through the urethra, and colonizing the bladder and kidneys (Figures 2, 3C). While UTIs in humans are prevalent among women in the community, catheter-associated UTIs afflict both genders equally and can cause serious adverse effects, as well as prolong hospital stays and health-related expenses. In the last 10 years, an alarming rise in multi-drug resistant isolates, especially of the sequence type (ST) 131 has further complicated treatment strategies as discussed in the next section (Coque et al., 2008; Nicolas-Chanoine et al., 2008; Totsika et al., 2011).

Studies utilizing human bladder cell lines and the murine infection models (Section Additional Reservoirs and Research Models: Murine Model) have revealed that during the initial stages of infection UPEC use type 1 pili to bind to uroplakin and integrins on superficial umbrella cells that line the bladder (Yamamoto et al., 1990; Connell et al., 1996a; Mulvey et al., 1998; Martinez et al., 2000; Mellata et al., 2003; Klemm and Schembri, 2004; Eto et al., 2007). These same pili are subsequently used to form intracellular bacterial communities (IBCs), which are biofilm-like structures within the bladder cell during early and middle stages of infection, between 2 and 8 h in the C3H/HeN and C3H/HeJ mouse models (Anderson et al., 2003; Justice et al., 2004; Hannan et al., 2012). Responding to yet uncharacterized signals, UPEC can egress from the transient intracellular state by filamenting and fluxing out of the infected host cell (Justice et al., 2004). The bladder cell can alternatively trigger an apoptotic-like cell death (Mulvey et al., 1998; Nagamatsu et al., 2015) and become exfoliated prior to UPEC filamentation, shedding the IBC into the bladder lumen. Bladder cell exfoliation exposes underlying host cell layers to invasion by UPEC that can remain quiescent for prolonged periods of time (Mulvey et al., 2000, 2001; Mysorekar and Hultgren, 2006). Quiescent intracellular reservoirs (QIRs) can cause recurrent infections (Mulvey et al., 2001; Figure 2). UPEC cells egressing from non-exfoliated bladder cells can re-initiate infection by engaging neighboring, naïve bladder cells, or by ascending to and colonizing the kidney. Studies with murine mouse models of UTI have also elucidated that UTI leads to urothelial remodeling and may fail to regenerate even weeks after treatment. RNA-seq revealed that immune-related pathways, as well as pathways pertaining to tissue morphology, cellular development, and cellular growth and proliferation were significantly enriched (Hannan et al., 2012; O'Brien et al., 2016).

In addition to type 1 pili, curli amyloid fibers, and P pili are critical for pathogenesis (Svenson et al., 1983; Dodson et al., 1993; Schilling et al., 2001; Barnhart and Chapman, 2006), along with other potentially uncharacterized adhesive fibers. UPEC strains can harbor more than one dozen different types of CUP pili, each with distinct adhesion specificities and differential regulation patterns (Welch et al., 2002; Chen et al., 2006; Spurbeck et al., 2011). The function and regulation of these fibers during UTIs are beginning to be elucidated (Spurbeck et al., 2011). In addition to adherence factors, iron acquisition is critical for UPEC pathogenesis, as is for almost all bacterial infections (Henderson et al., 2009; Cassat and Skaar, 2013).

Similar to APEC and NMEC, the presence of K1 capsule plays a role in pathogenesis during IBC formation (Anderson et al., 2010), while several toxins, such as hemolysin A, have been associated with fine-tuning host cell exfoliation during infection. Several recent studies have revealed that despite being facultative anaerobes, UPEC require aerobic respiration during acute UTIs (Alteri et al., 2009; Hadjifrangiskou et al., 2011; Floyd et al., 2015, 2016), indicating the presence of oxygen-sensing mechanisms that modulate virulence by production of type 1 pili, and possibly other factors, in response to altered oxygen levels. Furthermore, Shepherd et al. demonstrated that in addition to aerobic respiration, cytochrome *bd* oxidase is required for alleviating nitrosative stress during infection in the hypoxic bladder (Shepherd et al., 2016).

## Treatment strategies against ExPEC infection

Currently, there are no approved human vaccines against ExPEC; however, vaccines are used in farming practices against *E. coli* (Sadeyen et al., 2015). ExPEC infections in all afflicted populations are typically treated with antibiotics. Although this practice has been effective for many years both in the healthcare setting and in the poultry/farm industry, overuse, and misuse of antibiotics in the twentieth century has led to the emergence of multi-drug resistant ExPEC strains that are extremely difficult to eradicate. The recently emerged antibiotic resistant ST131 isolate harbors the *bla*_CTX−M−15_ gene producing extended spectrum beta-lactamases. ST131 isolates also harbor H4 serotype flagellar antigen, which augments adherence and invasion of bladder cells, and stimulation of IL-10 (Kakkanat et al., 2015). In addition to the multi-drug resistant ST131 isolates, cases of colistin-resistant uropathogenic and avian pathogenic *E. coli* are also emerging (McGann et al., 2016; Lima Barbieri et al., 2017). The first United States report of a colistin-resistant *E. coli* was released in early 2016, with an ST457 urine isolate from a Pennsylvanian woman with a UTI (McGann et al., 2016).

In the farm/poultry industry, a classic example of antibiotic resistance emergence is highlighted in the Yeruham study (Yeruham et al., 2006): a short 3-day antibiotic treatment resulted in many recurrent cases of UTIs in cattle, indicating the presence or the emergence of a resistant ExPEC population (Yeruham et al., 2006). In addition, the administration of antibiotics to farm animals increases the likelihood for asymptomatic colonization of animals by multidrug resistant ExPEC that can then colonize humans who come into contact with the cattle. Finally, thought-provoking studies by the Blaser group and colleagues are beginning to elucidate possible correlations between antibiotic use in farm animals and increasing obesity in humans (Blaser and Falkow, 2009), raising concerns about continued use of antibiotics in livestock.

Combined, these concerns are beginning to shift the focus of current research, not only to the development of new antibiotics, but also to the generation of agents that will have anti-virulence potential by targeting bacterial behavior as opposed to bacterial viability. For such agents to be effective, information about how bacteria, such as ExPECs, behave in response to environmental stimuli is crucial. Perhaps the most critical element in successful colonization and persistence in a specific niche is the ability of a pathogen to appropriately coordinate production of relevant virulence factors. This regulation must occur simultaneously with repression of other genes, the products of which are not needed in the particular environment. Bacteria are constantly bombarded with changing stimuli from within and outside the host. Within the host, these stimuli may come from innate immune defenses such as bursts of reactive oxygen species, cationic polypeptides and metal sequestration, as well as exogenous stressors such as antibiotics. In addition, the different stages of each infection cascade are accompanied by niche-specific changes in oxygen levels, nutrients, osmolality, and temperature. Each of these cues is sensed by one or more bacterial signaling systems that will then coordinate bacterial behavior. Below, we provide an overview of the signal transduction networks identified as important for the pathogenesis of NMEC, APEC, and UPEC focusing on TCSs.

## Two-component system signaling networks involved in ExPEC pathogenesis

Although eukaryotic-like serine/threonine kinases (Lux and Shi, 2005) are found within bacterial species, the majority of TCSs receptors are histidine kinases (Stock et al., 1989, 2000; Bijlsma and Groisman, 2003). TCSs encompass the predominant method by which bacteria sense and respond to the many different environments they encounter (Bourret et al., 1989; Stock et al., 1989, 2000). Prototypical TCSs comprise a membrane-embedded bacterial signaling receptor that is responsible for intercepting one or more specific stimuli or ligands. Signal transduction from the membrane-embedded receptor to the response regulator occurs via a phosphorelay event to a cognate partner protein, termed the response regulator, that will carry the output response (Bourret et al., 1989; Stock et al., 1989, 2000; Igo et al., 1990). The response regulator almost always resides in the cytoplasm and, in the majority of documented examples, acts as a transcriptional regulator (Stock et al., 1989, 2000).

Histidine kinases typically function as dimeric membrane receptors and consist of a sensing domain, a kinase domain, and a catalytic domain that binds and hydrolyzes ATP. Upon signal interception, the histidine kinase hydrolyzes ATP and undergoes auto-phosphorylation at a conserved histidine residue within the kinase domain (Figure 4). Histidine kinase auto-phosphorylation stimulates the transfer of the phosphoryl group to a conserved aspartate residue on the cognate response regulator (Figure 4), thus activating function.

Many sensor histidine kinases are bi-functional, having the ability to also act as phosphatases or reverse phosphotransferases, dephosphorylating the response regulator. Response regulator de-phosphorylation by the cognate sensor histidine kinase “resets” the signaling cascade, allowing the bacteria to respond again to the same stimulus upon re-exposure. Swift de-phosphorylation of the response regulator by the cognate histidine kinase also prevents aberrant activation of the response regulator by non-cognate kinases or other phosphor-donor molecules. Not much work has been performed in delineating the role of TCSs in ExPEC pathogenesis. Of the 62 conserved TCSs genes harbored by *E. coli* strains (Capra and Laub, 2012), only a handful have been studied in the context of pathogenesis. Notably, there also are strain-specific TCSs, harbored only by certain strains or pathotypes, which are of particular interest, such as the KguRS TCS (Cai et al., 2013). Below, we discuss key TCSs that have been shown experimentally to be important for UPEC, APEC, or NMEC pathogenesis (Figure 3).

### EnvZ-OmpR

The prototypical TCS, owing to the early characterization, is the OmpR-EnvZ system (Hall and Silhavy, 1981). The sensor kinase EnvZ is phosphorylated under hypo-osmotic conditions and phosphotransfers to the response regulator OmpR. Activation of OmpR leads to upregulation of outer membrane porin proteins, such as OmpF or OmpC (Igo and Silhavy, 1988; Forst et al., 1989; Cai and Inouye, 2002). OmpR has been shown to influence the expression of type 1 pili through transcriptional regulation of *fimB*, one of the site-specific recombinases that control the orientation of the type 1 pili promoter (Gally et al., 1996). In a murine model of UTI, deletion of *ompR* in the UPEC clinical isolate NU149 had a significant, two-log reduction in colony forming units in both the bladder and kidney, indicating a role of EnvZ-OmpR in pathogenesis (Schwan, 2009). UPEC encounter a significant change in extracellular osmolality as they exit the gut and ascend the urethra, so one can extrapolate that EnvZ-OmpR function is important during the early stages of infection (Schwan, 2009).

### CpxAR

The CpxAR system is comprised of the sensor kinase CpxA and the response regulator CpxR and is one of the *E. coli* systems responsible for sensing and coordinating the response to cell envelope stress (Pogliano et al., 1997; Raivio and Silhavy, 1997). Notably, the CpxAR system was one of the three TCSs shown to be indispensable for *E. coli* fitness in the murine gut (Lasaro et al., 2014). In UPEC, CpxAR has been shown to play multiple roles in pathogenesis. Originally identified by the Silhavy group, CpxAR activation was shown to occur upon binding of commensal *E. coli* to hydrophobic surfaces. CpxAR activation was shown to depend on the presence of the outer membrane lipoprotein NlpE, and this was the first demonstration of a function for NlpE (Otto and Silhavy, 2002). A follow up study revealed that activation of the Cpx system can occur in an NlpE-independent manner by inducing cues other than surface attachment (DiGiuseppe and Silhavy, 2003). CpxAR was also shown to sense and respond to misfolded pilin subunits during the assembly of P pili, which are adorned with the adhesin protein PapG that binds glycolipid receptors on urothelial cells lining the kidney (Hung et al., 2001; Lee et al., 2004). Joint collaborations from the Silhavy and Hultgren groups showed that the N-terminal extension of the PapE pilin subunit activated CpxAR (Lee et al., 2004). Mis-folded PapE and PapG also activate the CpxAR system; upon activation, the periplasmic protein CpxP is upregulated to alleviate membrane stress by guiding mis-folded proteins to be degraded by proteolysis (DiGiuseppe and Silhavy, 2003; Isaac et al., 2005). Most recently, the CpxAR system has been implicated in responding to antibiotics by altering the membrane integrity and increasing antimicrobial resistance (Raivio et al., 2013). CpxA has also been shown to sense high osmolality conditions and result in the repression of curli expression, an important component in the production of biofilms (Hou et al., 2014).

In UPEC, deletion of *cpxAR* impairs UPEC colonization of the murine bladder (Debnath et al., 2013). More recent studies demonstrated that CpxAR regulates expression of α-hemolysin (HlyA), though only about 50% of UPEC isolates encode this pore-forming toxin. HlyA causes cytotoxicity in urothelial cells. Nagamatsu et al. (2015) showed that loss of CpxR unleashes expression of HlyA and increases exfoliation of the host urothelium during infection, suggesting that CpxAR exerts a negative effect on *hlyA* expression, possibly fine-tuning cytotoxicity in urothelial cells for HlyA-harboring UPEC strains.

### RstAB

A recently described system thought to be involved in APEC pathogenesis, though relatively poorly understood to date, is the RstAB system. The RstA response regulator specifically appears to be important for APEC persistence in chicken macrophages and respiratory infection (Gao et al., 2015). RstAB is under the control of another TCSs called PhoPQ. The PhoQ sensor is activated in response to high levels of cationic polypeptides or low levels of magnesium, both of which are signals that have been suggested to directly interact with the PhoQ dimer (Miller et al., 1989; Prost et al., 2007). Expression of the *rstAB* gene pair has been shown to increase under low Mg^2+^ conditions in a PhoPQ-dependent manner (Minagawa et al., 2003). RstAB, in turn, modulates APEC survival under harsh conditions and adaptation to the extra-intestinal environment; deletion of the response regulator RstA shows decreased colonization of organs during systemic infection in chickens. Additionally, while the *rstA* mutant and the wild type parent were taken up by macrophages at similar levels, the *rstA* mutant could not persist within or escape the macrophages as well as the wild type strain (Gao et al., 2015).

### KguRS

A more recently discovered, primarily UPEC-encoded system is KguRS. The KguS sensor kinase was reported to sense the presence of α-ketoglutarate in the UPEC strain CFT073 (Cai et al., 2013). In a mouse model, deletion a CFT073 mutant deleted for *kguRS* colonized the urinary tract less efficiently. Given that α-ketoglutarate is primarily utilized in the tubules of the kidneys, the studies by Cai et al. implied that utilization of α-ketoglutarate enhances the ability of UPEC to adapt to the urinary tract environment (Cai et al., 2013).

### ArcA/B

The aerobic respiratory control system, or ArcA/B TCS, is a global regulator that facilitates adaptation from anoxic to aerobic conditions and mediates defense against reactive oxygen species (Loui et al., 2009). Unlike other TCSs, ArcB, and ArcA are not co-transcribed; ArcB is a tripartite sensor kinase that undergoes a phosphorelay event under anaerobic conditions. ArcA represses expression of many genes involved in aerobic respiration. In most cases, ArcA acts as a transcriptional repressor of enzymes involved in aerobic carbon metabolism. ArcA is a positive regulator of cytochrome d and pyruvate formate lyase involved in fermentation (Gunsalus and Park, 1994; Georgellis et al., 2001). Oxidation of cytosolic cysteine residues found within the ArcB histidine kinase, results in the formation of disulfide bonds, resulting in reverse phosphotransfer under aerobic conditions, de-activating ArcA (Georgellis et al., 2001; Morales et al., 2013). While most TCSs contain a large periplasmic sensing domain for the detection of stimuli, the short sensing domain of ArcB is necessary for detection of the physiological redox state of quinones in the electron transport chain in the cytoplasmic membrane (Georgellis et al., 2001). In APEC, loss of the ArcA response regulator severely attenuates virulence, due to loss of flagellar motility, chemotaxis, and proper metabolic function (Jiang et al., 2015).

### BarA-UvrY

While not located on the same operon, the BarA and UvrY proteins have been shown to function as a TCS (Pernestig et al., 2001). The BarA-UvrY TCS regulates the expression of the carbon storage regulation system, a master regulator between glycolysis and gluconeogenesis, which is necessary for bacterial function and long-term survival (Pernestig et al., 2003). The BarA (bacterial adaptive response) tripartite sensor is involved in protection from hydrogen peroxide stress through the activation of RpoS sigma factor. Functioning slightly differently from typical TCSs, tripartite sensors undergo a phosphorelay event: the phosphate group is transferred from the histidine residue to an aspartate residue to a second histidine residue, all of which are located in different domains of BarA, before transferring the phosphoryl-group to UvrY, the cognate partner (Suzuki et al., 2002). UvrY, while part of the *uvrYAC* operon has no apparent role in DNA repair (Suzuki et al., 2002). UvrY does, however, activate the CsrB protein, which increases the activation of biofilms. Deletion of BarA or UvrY results in a similar hydrogen peroxide hypersensitivity (Pernestig et al., 2001, 2003).

In a macaque cystitis model, competition profiles suggest that the BarA-UvrY TCS is crucial for the switch between different carbon sources present in the urine (Tomenius et al., 2006). In chicken embryos and in the murine model, UPEC strain CFT073 with a *barA* or *uvrY* deletion displayed reduced virulence through decreased production of hemolysin and LPS (Palaniyandi et al., 2012). Likewise, in APEC, the BarA-UvrY TCS has been shown to play a role in the chicken embryo infection model. Deletion of either *barA* or *uvrY* resulted in decreased expression of type 1 and P pili, diminishing adherence and persistence within embryonic tissues (Herren et al., 2006).

### QseBC

The QseBC system, comprised of the sensor kinase QseC and the response regulator QseB, was reported to be involved in quorum sensing in enterohemorrhagic *E. coli* (EHEC; Sperandio et al., 2002). EHEC QseC was shown to respond to norepinephrine, epinephrine, and autoinducer-3 (Clarke et al., 2006) and this deletion severely attenuates EHEC virulence (Hughes et al., 2009). In UPEC, deletion of *qseC* results in severe attenuation of UPEC due to reduced expression of motility genes, several CUP systems including type 1 pili, curli fibers, and several metabolic pathways (Kostakioti et al., 2009; Hadjifrangiskou et al., 2011). This misregulation of virulence factors occurs only in the absence of QseC, but not in the absence of QseB or the entire QseBC system. Recent studies have uncovered non-partner interactions that occur between the QseB response regulator and another TCS, PmrAB (Guckes et al., 2013). The PmrB sensor kinase of the polymyxin resistant (Pmr) AB TCS constitutively phosphotransfers to QseB in the absence of the QseC sensor (Guckes et al., 2013). This constitutive activation leads to aberrant gene repression by QseB and attenuation of virulence, making the QseBC system an excellent target for anti-virulence strategy development.

Interestingly, the PmrB sensor is known to respond to ferric iron and mediates alterations to the lipopolysaccharide (LPS) layer of the outer membrane to protect the cell against cationic polypeptide stress (Groisman et al., 1997; Wösten et al., 2000; Chen and Groisman, 2013). In wild-type strains of UPEC, elevated ferric iron, used as a proxy for cationic polypeptide stress, activates both PmrA and QseB response regulators in a PmrB-dependent manner, suggesting that in UPEC the PmrAB and QseBC systems naturally interact (Guckes et al., 2017). Understanding how these bacterial networks communicate during infection will elucidate new avenues for targeting bacterial virulence without applying selective pressure.

## Additional reservoirs and research models

### Zoonotic potential for urinary tract infection

Zoonotic transmission of ExPECs from animals to humans through the consumption of infected animal products is a newly identified route of transmission (Nordstrom et al., 2013). In addition to the typical, well-known ExPEC routes of transmission, recent studies have suggested the acquisition of an ExPEC infection through consumption of contaminated food products. One such example is the zoonotic potential of foodborne UTIs (FUTIs) in humans. A study published in 2015 shows that 129 out of 282 *E. coli* isolates sequence-typed as ExPEC strains. Status was determined by isolates containing 2 or more of the following ExPEC-associated genes: adhesins (*afaE8, bmaE, fimH, gafD, hra, papA, papC, papEF, papG, sfa*, and/or *focDE, sfaS*), toxins (*cdtB, cnf1, astA, hlyA, hlF, pic, tsh, sat*), siderophores (*fyuA, ireA, iroN, iutA*), protectins *(cvaC, iss, kpsM* K1, K2, and/or K100, *kfiC K5, rfc, traT*), and miscellaneous genes typically associated with extraintestinal *E. coli* (H7 *fliC, ibeA, ompT, malX, usp*; Mitchell et al., 2015). The *mcr-1* gene has also been isolated from *E. coli* found in pigs and chicken raised for retail meat consumption (Liu et al., 2016). Increased antibiotic use in feed or antibiotic misuse in treating bacterial disease in farm animals will increase the likelihood of transmission of antibiotic resistant *E. coli*. While not harmful in the human intestine, these ExPECs may cause subsequent infections if or when they enter different niches.

### Murine model

Small rodents have been used as models for neonatal meningitis (Kim et al., 1992; Mittal et al., 2011; Wijetunge et al., 2014), as well as UTIs (Yasuda et al., 1994; Kao et al., 1997; Hung et al., 2009; Hannan et al., 2012). In the meningitis model, 3-day old mice or 5-day old rats are orally inoculated with bacteria and are then followed over time (Mittal et al., 2010; Lemaître et al., 2014). In these models, animals become increasingly lethargic and show clinical signs of systemic infection, such as weight loss and behavioral abnormalities (Mushtaq et al., 2004). UTI models use 7–9 week old female mice, which are transurethrally inoculated with UPEC and followed over time (Hung et al., 2009). Murine models have been used to track the acute stages of UTI, using colony-forming unit (CFU) analyses, microscopy and immunological analyses (Hung et al., 2009; O'Brien et al., 2016); sub-acute stages and multi-strain infections (Alteri et al., 2015); or chronic, recurrent or catheter-associated phenotypes (Mysorekar and Hultgren, 2006; Hannan et al., 2010; Guiton et al., 2012). Studies have also used 7–8 week or older female mice as a menopause model to study UPEC infections (Wang et al., 2013; Kline et al., 2014).

## Concluding remarks

While TCSs are not the only sensory mechanism in bacteria, they provide a great infrastructure for signal detection and bacterial response. Combined with other signal detection mechanisms, TCSs modulate differential gene expression in response to the microenvironment surrounding the bacteria. The expanding genomic and transcriptomic/proteomic data are demonstrating a striking diversity in the extent and the kinetics of virulence factor expression, even among different strains of the same ExPEC pathotype. This is partly due to additional genetic elements that may impact the expression, abundance, or activity of a particular signaling system. For example, while epinephrine has been shown to serve as an activating signal for EHEC QseBC system (Clarke et al., 2006), the UPEC QseBC TCS does not respond to epinephrine, but becomes engaged in response to PmrAB activation via ferric iron (Guckes et al., 2013, 2017). Combined with the different stresses encountered in a niche-specific manner, these differences may be a function of different genetic elements between the GI and ExPEC pathotypes. Slight nuances in signaling between or within strains can alter the pathogenesis. Future studies may need to look at how strains that contain certain combinations of virulence factors are regulated and behave *in vivo*. Understanding the genetic profile and mechanisms of infection will help to generate anti-virulence therapeutics that do not kill bacteria, but rather re-wire their expression, allowing their recognition and elimination by host immune defenses.

## Author contributions

EB conceived the concept, performed the literature search and wrote the manuscript. AE assisted with the literature search and wrote the manuscript. MH oversaw the process, fact-checked the work and edited the manuscript.

## Funding

MH is supported by NIH NIAID 5 R01 AI107052-02. EB is supported by National Science Foundation Graduate Research Fellowship Program under Grant Number 1445197 and T32 GM07628 grant.

### Conflict of interest statement

The authors declare that the research was conducted in the absence of any commercial or financial relationships that could be construed as a potential conflict of interest.
